# Immune Circuits to Shape Natural Killer Cells in Cancer

**DOI:** 10.3390/cancers13133225

**Published:** 2021-06-28

**Authors:** Irene Mattiola

**Affiliations:** 1 Laboratory of Innate Immunity, Department of Microbiology, Infectious Diseases and Immunology, Charité-Universitätsmedizin Berlin, Campus Benjamin Franklin, Hindenburgdamm 30, 12203 Berlin, Germany; irene.mattiola@charite.de; 2 Berlin Institute of Health (BIH), Anna-Louisa-Karsch Strasse 2, 10117 Berlin, Germany; 3 Mucosal and Developmental Immunology, Deutsches Rheuma-Forschungszentrum, Charitéplatz 1, 10117 Berlin, Germany

**Keywords:** NK cells, tumor microenvironment, cancer

## Abstract

**Simple Summary:**

Natural killer (NK) cells are circulating innate lymphocytes endowed with antitumoral functions. NK cells are the innate counterpart of effector T cells and among the first cells responding to infections and tumors. In this review, the immune circuits regulating the NK cell antitumoral functions and the possible strategies to shape natural killing in cancer will be discussed.

**Abstract:**

Natural killer (NK) cells are innate lymphoid cells playing an important role in anti-cancer immunity. NK cells are efficient in controlling the spreading of metastasis but are not very powerful in fighting against primary tumors. The NK cell capability to infiltrate and persist in the tumor microenvironment and to exert their antitumoral functions is often limited by tumor escape mechanisms. These tumor-mediated strategies not only induce NK cell tolerance but also interfere with the NK cell-dependent immune networking. This review will provide an overview of the tumor escape mechanisms impacting NK cells, identify the immune circuits regulating the NK cell-dependent antitumor immunity and revise the emerging therapeutic approaches to unleash NK cells in cancer.

## 1. Introduction

Natural killer cells were discovered in the 1970s as large granular lymphocytes showing a spontaneous reactivity against tumor cells [1,2,3,4]. Similar to T cells, NK cells mediated the killing of target cells by the release of cytotoxic granules containing perforin and granzymes. NK cells did not express a T cell receptor (TCR) or any other receptors that use somatic recombination to create diversity and did not need antigen presentation for their activation. Therefore, NK cells were defined as innate lymphocytes. The activation of NK cells is mediated by a balance between activating and inhibitory signals, regulated by two general mechanisms: the “missing self-recognition” and the “induced cell recognition”. The NK cell inhibitory receptor repertoire endows NK cells with the recognition of MHC class I molecules, preserving the “self” from the natural killing. As virus-infected or tumor cells downregulate the expression of MHC I, they become a target for NK cells. On the other hand, virus-infected or tumor cells also express ligands for NK cell activating receptors. The binding of NK-cell-activating receptors by their ligands on target cells leads to the activation of NK cells through the “induced cell recognition”.

Until recently, we believed that innate lymphocytes consisted of only NK cells. The discovery of lymphoid tissue inducer (Lti) cells [5], group 1 (ILC1), group 2 (ILC2) and new subsets of group 3 innate lymphoid cells (ILC3) extended our idea of innate lymphocytes [6]. All innate lymphoid cell subsets lack the recombinant activating gene (RAG)-dependent rearranged antigen receptors, do not express myeloid cell phenotypical markers and show a lymphoid morphology. However, the different ILC populations are endowed with different functions, dictated by the expression of different transcription factors and different cytokine signatures [7]. NK cells represent the only cytotoxic ILC population, whereas the other groups of ILC are defined as helper-ILCs. Most of the literature on NK cells in cancer has been obtained before the discovery of the helper-ILCs. This calls for some critical thinking in interpreting some of the data included in this review. It is indeed quite difficult to discern NK cells from ILC1 or ILC1-like ILC3 in tissues [8] and therefore to distinguish between the NK cell and ILC1/ILC3-mediated antitumoral functions in tumors [9,10,11]. Certain antitumoral functions assigned to NK cells in the past might have been indeed the result of a joint effort between NK cells and the other helper-ILC subsets, unknown at that time [10].

Helper-ILCs are tissue-resident cytokine producer cells mainly located at barrier tissue. Conversely, NK cells are the most abundant subset of circulating innate lymphocytes [12,13]. Their circulating identity empowers NK cells to contain hematological malignancies [14] and to control the metastatic dissemination [15]. Although NK cells can be recruited to the tumor site, they are not very efficient in the eradication of primary tumors. This review will focus on the adverse environmental factors dampening the antitumoral functions of NK cells in solid tumors and on the immune circuits that shape the natural killing.

## 2. Adverse Environmental Factors Dampening the Antitumoral Functions of NK Cells

### 2.1. NK Cells in Metastases and Primary Tumors

Accumulating evidence shows that NK cells play a central role in the resistance to metastasis. As NK cells represent the major population of innate lymphocytes able to circulate in the blood (and lymphatic) stream, they take part in the first innate line of defense against disseminating metastasis [16]. In preclinical models, mice lacking NK cells or deficient in NK cell effector molecules showed a marked increase in the metastatic burden of syngenic tumor cells inoculated by different routes [15]. Although the direct delivery of cancer cells into the circulation is a useful experimental approach to obtain a rapid and consistent metastatic burden, it bypasses the early phases of metastatic dissemination and the influence exerted by the primary tumor on the metastatic niche. This limits our understanding of the contribution of NK cells to all the steps of the metastatic process. Studies in humans showed an inverse correlation between the abundance of circulating or primary-tumor infiltrating NK cells and the presence of metastasis at clinical presentation. This included patients with gastrointestinal sarcoma (GIST) [17], gastric, colorectal, renal and prostate carcinomas [18,19,20,21]. These findings ruled out any possible doubts on the fact that NK cells are indeed involved in the resistance to metastasis, despite the limitation of the preclinical models.

Conversely, the role of NK cells in the control of primary tumors remains a matter of debate. Although in the 1980s a couple of studies reported reduced tumor cell killing in individuals with defective NK functions [22,23], recent studies showed that NK cell-deficient patients were not more susceptible to cancer than the general population [24,25]. This, of course, fueled the idea that, indeed, NK cells were not effective in preventing the formation of primary tumors. However, low preoperative levels of NK cells in patients with colon carcinoma correlated with a higher risk of local recurrence upon tumor rejection [26], suggesting a tumor-protective role exerted by NK cells. Similarly, in breast cancer, the amount of tumor-infiltrating NK cells predicted the response to anti-HER2 antibodies, thus correlating with a good prognosis [27]. Therefore, some studies suggest a role for NK cells in antitumor immunity; however, conclusive evidence is still lacking.

Notably, NK cells were poorly represented in several types of primary tumors. NK cells were scarce in colorectal tumors [28] and, even if recruited to the tumor-draining lymph nodes, NK cells were almost absent in melanoma lesions [29,30]. NK cells were also the least abundant immune cell population infiltrating lung adenocarcinomas, and the few NK cells recruited to the tumor core were less cytotoxic compared to NK cells from normal lungs [31]. The low level of infiltration of NK cells in several tumors and the fact that the few tumor-infiltrating NK cells showed impaired effector functions argued for the presence of tumor escape mechanisms interfering with both the recruitment and the activation of NK cells in solid tumors.

### 2.2. Recruitment of NK Cells to the Tumor Site

The distribution of NK cells in our body is continuously changing. Although some NK cells can be found in tissues during homeostatic conditions, inflammatory cues boost the recruitment of circulating NK cells into tissues [32]. Recent evidence showed that NK cells preferentially populate highly vascularized organs such as the lung, the liver, the spleen, the pancreas and the kidney [32,33,34,35]. NK cells can also be found in the breast, the skin and the subcutaneous adipose tissue [13,32]. Infiltration of NK cells can be detected in hepatic, skin, pulmonary, breast and renal cancer [36]. Although the presence of NK cells in some of these tumors was associated with the patient overall survival [36], the frequency and the cytotoxic potential of the intratumoral and peritumoral NK cells in pulmonary, kidney and breast cancer was reduced compared to the healthy area of the same tissues [32,37]. These findings suggest that the part of the tissues where the tumor resides represents an “NK cell escaping” area compared to the healthy part of the same tissue.

**Figure 1:**
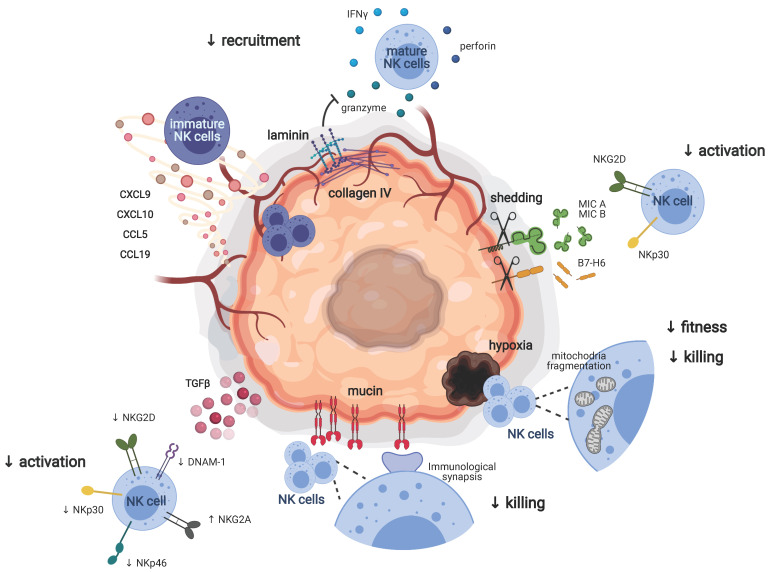
Tumor strategies to escape the natural killing. Primary tumors escape the antitumoral functions of NK cells by several mechanisms. On the one hand, tumors prevent the recruitment and infiltration of NK cells in the tumor core either by preferentially recruiting through a chemokine gradient an immature subset of NK cells (CD27^+^ CD11b^−^ in mice, CD56^bright^ in humans), which shows poor antitumoral functions and by interfering with the recruitment of a more mature and effector subset of NK cells (CD27^−^ CD11b^+^ in mice, CD56^dim^ in humans) by creating physical barriers built of laminin and collagen IV. On the other hand, tumors dampen NK cell activation and effector functions to escape the natural killing. Tumors shed ligands for NK cell activating receptors to avoid the recognition by NK cells and induce TGFβ-dependent downregulation of activating receptors and upregulation of inhibitory receptors on NK cells, strongly impacting their activation. Tumors also prevent the formation of the immunological synapses necessary for NK cell-mediated killing by creating a mucin protective layer. Finally, hypoxic areas in primary tumors induce the fragmentation of NK cell mitochondria, thus suppressing NK cell survival and their capability to eliminate tumor cells. Figure created with BioRender.

In humans, it has been reported that the NK cells that were infiltrating non-small cell lung carcinomas (NSCLC) and breast cancer preferentially showed a CD56^bright^ perforin^low^ phenotype compared to normal tissues [33,37]. This suggested selective recruitment of certain NK cell subsets to the tumor bed. By recalling specifically CD56^bright^ NK cells, which are considered as an immature population of NK cells with less cytotoxic potential compared to the CD56^dim^ population, pulmonary and breast tumors might protect themselves from NK cell killing. In accordance with this, NSCLC and breast cancer showed an upregulation of chemokines, such as CXCL9, CXCL10 and CCL19, acting on CCR7 and CXCR3, which are chemokine receptors mainly expressed by CD56^bright^ NK cells, whereas chemokines involved in the recruitment of the CD56^dim^ population such as CXCL2 and CX3CL1 were downregulated [33] (Figure 1). The same findings were applicable to murine tumors, where the immature population of NK cells expressing CD27 was more represented than the mature population of NK cells expressing CD11b [38] and our unpublished data. As observed in humans, the intratumoral accumulation of immature NK cells in RMA tumors was mainly driven by a gradient of chemokine selectively acting on CXCR3, highly expressed by CD27^+^ NK cells [38]. CCR5, the receptor for CCL5, is highly expressed by immature human and murine NK cells (CD56^bright^ and CD27^+^, respectively). In a preclinical model of melanoma, it has been shown that a gradient of CCL5 was needed for the recruitment of NK cells either to the primary tumor or to metastasis [39,40]. Although it is a chemokine mainly involved in the recruitment of monocytes, recent evidence showed that CCL2 was required for the recruitment of NK cells to IL17D-enriched melanoma. As CCR2 was mainly expressed in murine immature NK cells, the authors reported a preferential infiltration of the CD27^hi^ NK cell subset [41]. Taken together, these findings demonstrated that tumor cells established a chemokine gradient aimed to selectively recall the immature and less cytotoxic population of NK cells as part of their strategy to escape the natural killing (Figure 1).

The CD56^bright^ NK cells recruited to pulmonary cancer lesions are mainly located in the tumor stroma and at the interface between stromal and tumor cells. The few NK cells detected within the tumor core did not appear to be in direct contact with tumor cells [37,42]. Already in the late 1990s, it was suggested that high levels of collagen type IV and laminin in the extracellular matrix of certain tumors correlated with low infiltration of NK cells, thus suggesting a role of collagen IV and laminin in preventing NK cell invasion to the tumor core [36]. These observations argue for the presence of a further step of protection carried out by the tumor stroma that prevents NK cells from reaching the tumor core (Figure 1). Interestingly, these tumor escape mechanisms seemed to not apply to metastasis. Indeed, in contrast to what was observed in primary tumors, NK cells could be detected both in the center and in the periphery of human melanoma metastases. Sc-RNA seq analysis of NK cells isolated from the different areas of metastatic tumors (center, cortex and distinct nodule in the cortex) showed a certain extent of gene variability within the different areas and generated six different clusters. Interestingly, NK cells sitting in the nodule in the cortex expressed high levels of AREG, XCL1, XCL2 and FOS, all genes involved in the recruitment of DC. Conversely, NK cells located in the center and the cortex of the metastasis showed a more cytotoxic signature, characterized by the expression of granzymes and perforin [43]. These evidence may be indicative of the need for NK cells to be in close contact with the tumor cells in order to exert their killing functions. As part of a nascent tumor, metastases are not encapsulated in a well-organized matrix, and this may promote the infiltration of NK cells, the direct contact with tumor cells and the efficiency of the killing.

### 2.3. Tumor Escape Mechanisms Driving NK Cell Tolerance

NK cells infiltrating primary tumors are mainly characterized by a dysfunctional phenotype. Indeed, NK cells infiltrating several cancers, including gastric cancer and hepatocellular carcinoma, showed reduced IFNγ and TNFα production as well as reduced proliferation and cytotoxicity compared to NK cells from healthy tissues [44,45]. This suggested the presence of tumor escape mechanisms dampening the activation of the few NK cells able to reach the primary tumors.

#### 2.3.1. NK Cell Activating and Inhibitory Receptors

It is well-accepted that the activation of NK cells results from a balance between activating and inhibitory signals. To prevent NK cell activation, tumor cells evolved to express a pattern of NK cell inhibitory ligands, such as classical and non-classical MHC-I [46], and to downregulate the expression of NK-cell-activating ligands.

NKG2D is an activating receptor recognizing the cell surface glycoproteins MHC class I polypeptide-related sequence A (MICA) and B (MICB) and UL16 binding proteins (ULBP 1–6), which are induced by DNA damage, genotoxic or oxidative stress [47]. Using NKG2D deficient mice, Guerra et al. demonstrated that NKG2D was required for preventing the incidence and onset of spontaneous and transplantable tumor models [48]. However, to evade NK cell recognition and to reduce the NKG2D-mediated NK cell activation, tumor cells shed MICA and MICB from their surface [49] (Figure 1). The shedding of NKG2D ligands by tumor cells indeed leads to the downregulation of NKG2D on the surface of NK cells and T cells, further dampening the antitumor immunity [50,51].

Similar to the NKG2D ligands MICA and MICB, the NKp30 ligand B7-H6 could be cleaved from the surface of tumor cells and released as soluble B7-H6, thus preventing the NKp30-mediated recognition of tumor cells [52,53] (Figure 1).

NKG2D and NKp30 are not the only NK cell receptors playing a role in cancer. It has been demonstrated that the deletion of NKp46 led to an increased metastatic burden and reduced IFNγ and TNFα production by NK cells [54]. By the use of NKp46-Fc antibodies, it has been demonstrated that certain tumor cell lines and the primary tumor expressed NKp46 ligands [54,55]. These findings argued for a potential implication of NKp46 in tumor surveillance. However, further studies are required to identify the tumor-associated molecules inducing the NKp46-dependent activation of NK cell antitumoral functions.

Although recent data showed that the MHC class II variant HLA-DP could bind NKp44 and might impact NK cell activation upon viral infection [56]; there is no evidence demonstrating a role of NKp44 in cancer.

DNAM-1 is a cell surface glycoprotein expressed by NK cells and involved in tumor recognition. The ligands for DNAM-1 are CD112 and CD155, two poliovirus receptors (PVR) expressed on pathogen-infected or malignant cells [57]. As these molecules have the peculiar feature of binding both DNAM-1 and TIGIT, an inhibitory receptor on NK cells, the expression of CD112 and CD155 by tumor cells could potentially play a dual role either by triggering or dampening NK cell effector functions. Recent evidence showed that DNAM-1 deficiency or blockade in mice did not influence the immune cell control of syngeneic tumors [47,58]. Conversely, in several preclinical models of cancer, the blockade of TIGIT prevented NK cell exhaustion and promoted NK cell-dependent tumor immunity [47,58]. These findings suggested that CD112 and CD155 expressed by tumor cells might work better as inhibitory ligands of TIGIT rather than activating ligands of DNAM-1.

In breast and pancreatic cancer, it has been observed that the downregulation of NKp30, NKG2D, NKp46 and DNAM-1 accompanied by the upregulation of the inhibitory receptor NKG2A correlated with impairment of NK cell cytotoxicity. The blocking of TGFβ partially rescued the expression of these NK-cell-activating receptors [59], suggesting that soluble molecules in the tumor microenvironment could be responsible for the modulation of the NK cell receptor repertoire (Figure 1). Of note, TGFβ impacted not only the expression of activating receptors but also the expression of chemokine receptors, thus preventing NK cell recruitment to the tumor site. These evidence argued for a dual role of TGFβ in favoring NK cell escape mechanisms [60].

#### 2.3.2. Beyond NK Cell Receptors

Emerging evidence suggests that besides the dysregulation of NK cell activation and tumor cell recognition involving NK cell receptors, alternative strategies can be exerted by the tumor to impair NK cell antitumoral functions. Recently, Zheng et al. observed that human NK cell infiltrating liver cancers showed small and fragmented mitochondria compared to NK cells located out of the tumor. The fragmentation of the mitochondria correlated with reduced NK cell fitness and killing ability, leading to tumor evasion and bad prognosis (Figure 1). Notably, mitochondria fragmentation was mediated by the sustained activation of rapamycin-GTPase dynamin-related protein 1 (mTOR-Drp1) in NK cells as a consequence of the generation of a hypoxic tumor microenvironment [61]. Several solid tumors showed high oxygen consumption and disorganized vascularization leading to the generation of the area that undergoes transient or permanent hypoxia. Genes involved in the regulation of metabolic and biosynthetic processes were impacted by hypoxia as a result of their modulation through hypoxia-inducible transcription factors (HIF), which were indeed largely involved in the glycolytic metabolism [62]. These recent findings open new insight into the importance of NK cell metabolism in cancer.

NK cells need direct contact with tumor cells to exert natural killing. The formation of an immunological synapsis is indeed required for the release of the cytotoxic granules. It has been shown that ovarian cancer cells interfered with the formation of the immunological synapses by producing anti-adhesion molecules such as the glycoprotein mucin 16 [63] (Figure 1). This represents a further step of protection from NK cell killing.

#### 2.3.3. Therapeutic Approaches to Unleash NK Cells in Cancer

Several therapeutic approaches aimed to boost NK cell antitumoral functions and to overpass tumor escape mechanisms, either by enhancing NK-cell-activating receptors or by blocking the inhibitory ones, have been recently proposed. Ferrari De Andrade et al. developed a blocking antibody that, by targeting their a3 domain, prevented the shedding of MICA and MICB from tumor cells. This promoted not only the binding of NKG2D but also the engagement of Fc receptors on NK cells, enhancing their antitumor activity [64]. Gaulthier et al. have shown that multifunctional antibodies called NK cell tri-specific killer engagers targeting on one side NKp46 and CD16 expressed by NK cells and on the other side tumor antigens could create a bridge in between NK cells and tumor cells facilitating natural killing [65]. Zhang et al. demonstrated that a blocking antibody directed against TIGIT not only improved NK cell antitumor activity but also induced a potent NK cell-dependent tumor-specific T cell immunity, enhancing the efficiency of PD-L1 treatments. This finding opened new perspectives on the use of anti-TIGIT antibodies in anti-cancer therapeutic approaches [58]. Andrè et al. worked on inhibitory receptors and showed that in different preclinical models of cancer, the blocking of NKG2A expressed by both NK cells and T cells enhanced NK cell-mediated antitumor immunity. Interestingly, by combining anti-NKG2A and anti-PD-1, the efficiency of the anti-NKG2A antibody was extended to T cells [66]. Nath et al. found that NK cells expressed high levels of CD47 [67], an inhibitory signaling receptor for thrombospondin-1 mainly expressed by T cells. The blockade of CD47 correlated with increased NK cell proliferation and activation, leading to more efficient antitumor activity [68].

Adenosine is a hypoxia-induced immunosuppressive metabolite acting on both T cells and NK cells and is highly detectable in the tumor microenvironment [69,70]. Recent evidence suggested that adenosine impacts NK cell maturation. Indeed, mice deficient for the adenosine receptor A2AR showed enhanced NK cell maturation at steady-state. The conversion to a terminally mature stage correlated with an increased capability of NK cells to exert their effector functions, including antitumor immunity [71]. The development of “next generation” therapeutics inhibiting extracellular adenosine paved the ground for a possible innovative approach to interfere with the immunosuppressive milieu and to unleash NK cell killing [72].

Although NK-cell-activating cytokines in the tumor microenvironment contribute to tumor suppression, the same cytokines may promote the expression of immune checkpoints, ultimately suppressing NK cell function. For instance, the sustained persistence of IL-15 in the tumor microenvironment induced the expression of the IL-15-inducible inhibitor of IL-15-signalling, namely, cytokine-inducible SH2-containing protein (CISH). CISH bound the IL-15R complexes leading to its rapid degradation via the ubiquitin ligase-proteasomal pathways and rendering NK cells less responsive to IL-15 [73]. Similarly, IL1R8 expressed by mature and activated NK cells represented a negative regulator of IL-18 signaling and dampened NK cell antitumoral functions in NK cell-enriched tumors [74]. Therefore, neutralization of these cytokine-dependent NK cell checkpoints represents a suitable strategy to interfere with the negative feedback loop and to keep persistent NK cell effector functions in cancer.

Taken together, these evidence demonstrated that a deeper understanding of the mechanisms exerted by tumor cells to induce NK cell tolerance is of extreme importance for the discovery of potential therapeutic tools aimed to unleash NK cell antitumoral functions in primary tumors.

## 3. Plasticity of Innate Lymphoid Cells in Cancer

Whereas the deleterious effect of a suppressive tumor microenvironment on myeloid cells has been well established, the impact of the tumor microenvironment on the innate lymphoid cells has only recently emerged. It has already been described that certain pathological states, including IBD and airway inflammation, induced trans-differentiation between ILC1 and ILC3 or ILC2 and ILC1 [75,76]. The first evidence of such plasticity in cancer dates back to 2017 when two different groups reported that NK cells could revert to an ILC1-like phenotype in a preclinical model of cancer [77,78]. Interestingly, in both works, the trans-differentiation of NK cells to ILC1 was mediated by TGFβ signaling. Gao et al. observed that CD49a^−^CD49b^+^Eomes^+^ NK cell infiltrating sarcomas by upregulating CD49a and downregulating CD49b and Eomes acquired an ILC1-like phenotype characterized by the lack of the natural killing. This continuum between ILC1 and NK cells determined by a gradient of Eomes and CD49b recalls the canonical maturation steps of conventional NK cells, fueling the discussion of whether ILC1 might represent an immature state of NK cells [79,80]. Conversely to NK cells, CD49a^+^CD49b^+^Eomes^+^ (intILC1) and CD49a^+^CD49b^−^Eomes^int^ (ILC1-like) cells failed to control tumor growth and metastases, suggesting that the conversion between NK cell and ILC1 represented a tumor escape mechanism [77]. TGFβ was identified as the driving force of this conversion. Interestingly, the identity of NK cells and ILC1 was partially determined by a gene signature indicative of an “imprinting” by cytokines of the TGF-β family. SMAD4 is a signal transducer that facilitates the signaling pathway common to all the cytokines belonging to the TGF-β family. NK cells from mice lacking SMAD4 showed an ILC1-signature and were unable to control tumor metastasis or viral infections [78]. Therefore, in the presence of TGF-β, tumor-infiltrating NK cells could be converted to an ILC1-like phenotype lacking their ability to control tumor progression.

TGF-β is not the only molecule that triggers the SMAD pathway in NK cells. Activin-A binds to a set of receptors (different from the one bounded from the isoforms of TGF-β) that activate SMAD2/3 signaling. As TGF-β, also activin-A has been associated with a bad prognosis in cancer. In 2019, Rautela et al. showed that activin-A induced the expression of CD49a on splenic NK cells and suppressed the killing against tumor cells, even if at a lower extent compared to TGF-β. Interestingly, the blocking of activin-A reduced the metastatic burden in WT mice, suggesting that activin-A inhibitors could improve antitumor immunity and thus NK cell control of metastasis [81]. These data argued for possible involvement of activin-A in the conversion from NK cells to ILC1. However, further studies are required to better understand the role of activin-A in NK cell plasticity in cancer.

All these evidence convey a fascinating and reasonable concept of tumor-induced plasticity between group 1 innate lymphoid cells. However, the lack of fate-mapping tools limits the conclusions drawn from these studies. It is quite difficult to discern between NK cells and ILC1 in tissues, and this might be even more difficult in tissues with an increased level of complexity, such as primary tumors. Furthermore, we have to take into account that part of ILC1 is composed of ex-ILC3, as shown by Diefenbach and colleagues [82,83]. Ex-ILC3 are ILC3 that had expressed RORγt but did not express RORγt anymore. The lack of RORγt coincides with the gain of T-bet expression, making these cells closer to ILC1 than to ILC3. As ex-ILC3 express both NKp46 and NK1.1, the only way to distinguish ex-ILC3 from ILC1 is by fate-mapping of RORγt. Therefore, a certain grade of criticism has to be applied when we talk about innate lymphoid cell plasticity in the absence of fate-mapping.

However, the arrival of next-generation sequencing may contribute to identifying gene-specific signatures associated with certain tumor-infiltrating ILC subsets that might help to better discern between NK cells, ILC1 and ex-ILC3 in cancer. In a very recent publication, McFarland et al. used sc-RNA sequencing to elucidate gene signatures of mouse ILC1-NK cells from tissues, tumors and circulation [84]. They identified unique transcriptional programs to define circulating and tissue NK cells and tissue-resident ILC1, and they showed that the programs of tumoral NK cells were distinct from those of the other NK cells. This work represents the first evidence that by combining transcriptional programs and surface expression markers, it is indeed possible to extend our understanding of tumor-associated innate lymphocytes. However, in their analysis, McFarland et al. did not consider ex-ILC3. Therefore, further studies are required to complete the picture and fully address the complexity of group 1 innate lymphocytes in tumors.

## 4. Immune Cell Networking in the Tumor Microenvironment

Immune responses are the result of networking between immune cells. The presence of immunosuppressive cells in the tumor microenvironment jeopardizes the network and thus the success of the immune response against tumors. NK cells are innate cells whose antitumoral functions are mainly triggered by myeloid cells. In turn, NK cells orchestrate the myeloid and the adaptive antitumor immunity by producing immunomodulatory factors, such as IFNγ, TNFα and CCL5. The understanding of the cellular and molecular mechanisms underlying the failure or the success of NK cell networking in the tumor microenvironment is essential to identify potential targets to unleash not only the power of the natural killing but also the efficiency of the whole antitumor immune response. This section will focus on recently emerged immune circuits that are influencing the efficiency of NK cell antitumoral functions.

### 4.1. NK Cells and Platelets

**Figure 2:**
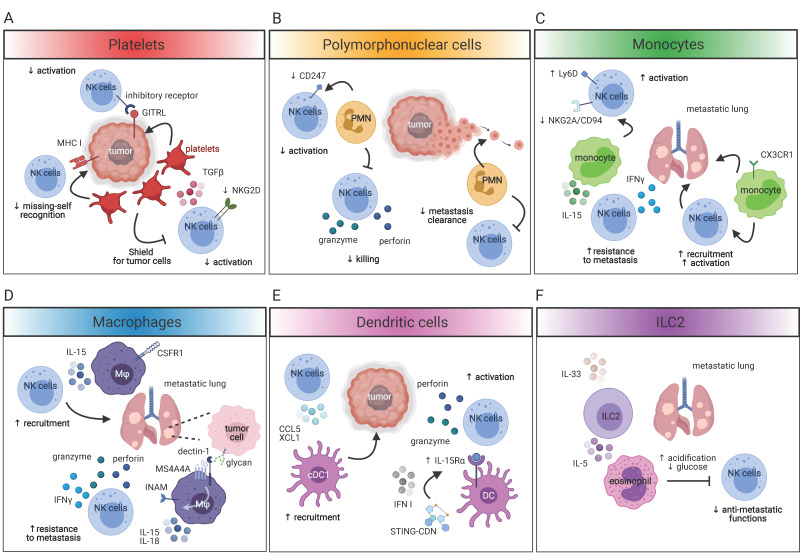
The innate networking of NK cells in the tumor microenvironment. NK cell antitumoral functions are strongly influenced by the cross-talk of NK cells with other innate immune cells in the tumor microenvironment. (**A**) NK cell activation is negatively regulated by platelets. Platelets promote downregulation of NK-cell-activating receptors and empowers tumor cells with NK cell inhibitory signals. (**B**) Polymorphonuclear cells actively participate in the metastatic dissemination by impairing NK cell activation and killing and reducing NK cell-mediated clearance of metastases. (**C**) Patrolling monocytes sustain NK cell activation by maintaining high levels of NK cell activating receptors and low levels of NK-cell inhibitory receptors and improve the NK-cell-mediated resistance to metastasis by triggering NK cell IFNγ production via the release of IL-15. CXCR1^+^ monocytes also play an important role in the recruitment of NK cells to the metastatic site. (**D**) MS4A4A- and CSFR1-expressing macrophages induce NK cell recruitment and activation through the release of IL-15 and IL-18, promoting NK cell resistance to metastasis. (**E**) STING-CDN induce the expression of IL-15R in DC via IFN-I, favoring the trans-presentation of IL-15 to NK cells and thus the activation of NK cell antitumoral functions. On the other hand, tumor-infiltrating NK cells promote the recruitment of cDC1 to the tumor bed by releasing CCL5 and XCL1. (**F**) In the presence of IL-33, pulmonary ILC2 induces the recruitment of eosinophils to the metastatic site by releasing IL-5. Eosinophils promote the acidification of the metastatic microenvironment by reducing the levels of glucose. Therefore, they compromise NK cell metabolism and fitness and, ultimately, the ability of NK cells to control the metastatic burden. Figure created with BioRender.

Platelets are thrombocytes that limit blood loss and promote wound healing. Growing evidence suggests that platelets are active players for cancer progression, favoring cancer cell extravasation and metastasis [85]. It has been reported that platelets work as a shield for tumor cells, protecting them from TNF and NK cell-induced cell death [86,87]. The shielding of tumor cells by platelets was mediated either by membrane glycoproteins which interact with tumor cell integrins, and by the binding of P-selectin expressed by platelets with mucins on the surface of tumor cells [88] (Figure 2A). Platelets are also endowed with TGF-β production, thus dampening NK cell maturation and activation and impairing NK cell recognition of tumor cells. Indeed, platelets can interfere with the NKG2D/NKG2DL axis by inducing either the TGF-β-mediated downregulation of NKG2D on NK cells or the shedding of NKG2D ligands from tumor cells [89,90] (Figure 2A). Furthermore, platelets can transfer “normal” MHC I molecules onto the surface of tumor cells, impairing the “missing-self” recognition by NK cells or inducing the upregulation of NK cell inhibitory ligands, such as glucocorticoid-induced TNF-related ligands (GITRL), on tumor cells, leading to NK cell tolerance [91,92] (Figure 2A). The fact that thrombocytopenia reduced the metastatic burden in mice in a NK cell-dependent manner further argued for an active role of platelets in promoting the dissemination of metastasis by interfering with NK cell antimetastatic functions [93].

### 4.2. NK Cells and Myeloid Cells

Monocytes, macrophages, dendritic cells [94] and neutrophils constitute the major immune infiltrate in most tumors and, along with NK cells, are likely among the earliest immune effectors within the tumor microenvironment [47].

#### 4.2.1. Polymorphonuclear Cells

Whereas the cross-talk between MDSCs and T cells has been largely studied, the networking between NK cells and MDSCs has been poorly explored [95]. For definition, MDSCs are TGF-β-producing cells [96,97]. TGF-β has important NK cell inhibiting functions in cancer [77,78]. This argues for a potential role of MDSCs in dampening NK cell antitumoral functions. However, more accurate studies are required to better elucidate the TGF-β dependent cross-talk between NK cells and MDSCs. What is known, though, is that MDSCs can modulate NK cell antitumoral functions by targeting NK-cell-activating receptors. It was reported that MDSCs induced the downregulation of CD247 expression on the surface of NK cells. CD247 is a subunit required for the signaling of Natural Cyototoxicity Receptors (NCRs) and CD16. Therefore, the downregulation of CD247 correlated with impaired NK cell activation [98,99] (Figure 2B). In vitro data also suggested that MDSCs inhibited NK cell effector function through the engagement of NKp30 [100]. However, further studies are required to better dissect how MDSCs could engage NKp30 and if this could correlate with NK cell tolerance. In a preclinical model of breast cancer, Spiegel et al. showed that neutrophils promoted the extravasation of intraluminal tumor cells, protecting them from NK cell-mediated clearance and thus promoting metastatic dissemination [101] (Figure 2B). Sceneay et al. found that CD11b^+^Ly6C^med^Ly6G^+^ cells recruited to hypoxic breast tumors or melanoma compromised NK cell cytotoxicity in the premetastatic niche [102], thus increasing metastatic potential. Taken together, these findings clearly pointed to the role of neutrophils in dampening NK cell antitumoral functions. However, in very specific contexts, MDSCs can also enhance NK cell effector functions. Nausch et al. showed that MDSCs infiltrating lymphomas expressed NKG2D ligands, and thus were able to activate NK cells [103]. Importantly, these MDSCs were still efficient in suppressing T cells, confirming their identity as suppressor cells. Of note, the authors defined MDSCs as CD11b^+^Gr-1^+^F4/80^+^. The expression of F4/80 in this population might suggest a monocyte/macrophage origin of these cells rather than a polymorphonuclear one.

#### 4.2.2. Monocytes, Macrophages and DC

Tumor-infiltrating NK cells are activated by direct recognition of tumor cells or by pro-inflammatory cytokines, including IL-12, IL-15 and IL-18 [104]. NK cell-activating cytokines are mainly produced by activated monocytes, macrophages and dendritic cells [104]. Several pathways in the tumor microenvironment induce the release of NK-cell-activating cytokines. Cell death generates immunogenicity mediated by danger signals [105]. Tumor debris and DNA in the tumor microenvironment contain damage-associated molecular patterns (DAMPs) that trigger the activation of monocytes, macrophages and dendritic cells by acting on pattern-recognition receptors [105]. Recently, it has been shown that tumor cells express certain carbohydrate moietis (i.e. N-glycans) that are working as tumor-associated molecular patterns (TAMPs) and thus are able to induce the release of NK-cell-activating cytokines by macrophages and dendritic cells, in particular, in the context of metastasis [106,107]. As they strongly promote the priming of NK cells, NK-cell-activating cytokines produced by myeloid cells reduce the threshold required for NK cell activation by tumor ligands, and high levels of such cytokines were correlated with favorable outcomes [47].

Monocytes are circulating cells that can be rapidly recruited to the tumor microenvironment, where they are activated and produce high levels of pro-inflammatory cytokines. Few publications reported the existence of a cross-talk between monocytes and NK cells in cancer. It is known that IL-15 produced by splenic monocytes promotes the differentiation and thus the effector functions of splenic NK cells, arguing for a role of monocytes in enhancing NK cell antitumoral functions [108]. In line with this, Hanna et al. showed that patrolling monocytes favored the recruitment and activation of NK cells to metastatic lungs in different preclinical models of cancer. The authors demonstrated that patrolling monocytes were actively recruited to the metastatic sites in a transplantable model of lung carcinoma, melanoma and in a spontaneous model of breast cancer. This accumulation of patrolling monocytes was dependent on CX3CR1. Patrolling monocytes reaching the metastatic sites scavenged tumor debris and were involved in the recruitment and activation of NK cells, thus participating in the control of metastasis [109] (Figure 2C). This shed new light on the role of patrolling monocytes in promoting antimetastatic functions of NK cells. These findings were further extended by Kubo et al. They found that in the presence of a primary melanoma, an increased number of NK cells expressing a high level of IFNγ, perforin and granzyme were recruited to the metastatic lung. This accumulation of activated NK cells in the lung of tumor-bearing mice was mirrored by an increase in patrolling monocytes and macrophages expressing iNOS and MHC II. Interestingly, IL-15 produced by patrolling monocytes in response to primary tumors was responsible for the induction of NK cell-derived IFNγ and thus required for the resistance to metastasis [110] (Figure 2C). The Hedrick group also showed that in the absence of patrolling monocytes, NK cells recruited to metastatic melanoma sites in the lung showed decreased expression of the activating receptor Ly49D and increased expression of the inhibitory receptor NKG2A/CD94. This correlated with reduced cytotoxicity against tumor cells, further confirming that patrolling monocytes are indeed required for NK cell recruitment, activation and antimetastatic functions [111] (Figure 2C). Taken together, these evidence demonstrated that monocytes are inducers of NK cells’ antimetastatic functions through the production of pro-inflammatory cytokines, namely, IL-15. However, whether monocytes play the same role in the microenvironment of primary tumors is still not known.

The concept that tumor-associated macrophages are dampening NK cell activation by exerting suppressive functions has been revised, particularly in the context of metastasis. Recently, it has been demonstrated that MS4A4A, a tetraspan molecule highly expressed by tumor-associated macrophages, was required for proper activation of NK cells in a preclinical model of metastatic melanoma and colon-carcinoma. MS4A4A is a tetraspan-like molecule able to interact with certain immune receptors and to regulate their signaling pathway. By interacting with Dectin-1 on macrophages, MS4A4A enhanced the Syk dependent pathway and ensured the release of pro-inflammatory cytokines, including IL-15 and IL-18. MS4A4A^+^ macrophages expressing Dectin-1 recognized specific carbohydrates moieties (N-glycans) expressed on tumor cells, leading to the triggering of the Syk pathway downstream Dectin-1 and thus the production of pro-inflammatory molecules required for the activation of NK cell-mediated cytotoxicity, IFNγ and resistance to metastasis [107] (Figure 2D). Therefore, although tumor-associated macrophages promote cancer, they may play an opposite role when it comes to metastasis expressing high levels of glycans. This NK cell-mediated antitumoral role of metastasis-associated macrophages is in line with other studies. Beffinger et al. showed that in a preclinical model of melanoma, CSFR1 blockade diminished the number of NK cells due to the reduction in IL-15-producing macrophages (Figure 2D). This correlated with an increased seeding of metastatic tumor cells in the lung. Interestingly, the combined administration of IL-15 with CSFR1 inhibitor mitigated the indirect effect of the CSFR1 inhibitor on NK cells. These studies demonstrated that IL-15 produced by macrophages was essential for the clearance of metastatic cells by NK cells [112].

The cross-talk between NK cells and DC has been extensively studied in the past, both in homeostatic and pathological conditions. As the networking between NK cells and DC in cancer has been recently reviewed [113], this review will focus only on a couple of recently emerged pieces of evidence. Nicolai et al. demonstrated that in vivo administration of STING-activating cyclic dinucleotides (CDN) induced an IFN I-dependent upregulation of IL-15Rα on the surface of DC promoting NK cell activation, cytotoxicity and antitumoral functions in several preclinical models of cancer [114] (Figure 2E). Of note, the NK cell-mediated rejection of tumor was independent of CD8 T cells. Although it was already known that DC trans-presentation of IL-15 was required for NK cell priming in sterile inflammation [115], the possibility to trigger this pathway by immune-modulating agents in the context of primary cancer may represent an innovative approach for cancer immunotherapies. Böttcher et al. showed that the cross-talk between NK cells and DC in cancer is bidirectional. Indeed, by secreting CCL5 and XCL1, NK cells favored the recruitment of cDC1 in the tumor microenvironment, promoting immune control of melanoma (Figure 2E). Notably, this pathway was efficient only in the absence of tumor-produced PGE_2_ [116]. Indeed, PGE_2_, by acting on EP2 and EP4 on NK cells, impaired their capability to release IFNγ and thus to maintain a cancer inhibitory microenvironment [117]. Therefore, the targeting of PGE_2_ in tumors might unleash a NK cell/DC cross-talk and activate anti-cancer immunity. It is still not known though, whether these cDC1 recruited to the tumor could, in turn, sustain NK cell antitumoral functions.

All together, these evidence suggest for innovative therapeutic approaches to improve antitumor immunity by boosting the cross-talk between NK cells and DC in cancer.

### 4.3. NK Cells and Helper-Innate Lymphoid Cells

NK cells are not the only innate lymphocyte infiltrating the tumor microenvironment. The presence or absence of other innate lymphoid populations in the tumor microenvironment could depend on the tissue where the tumor onsets. Therefore, the networking of NK cells and the other innate lymphocytes can vary depending on the tissue of origin of the tumor.

Due to the difficulties in discerning between tumor-infiltrating NK cells, ILC1 and ex-ILC3, the investigation of the cross-talk between these innate lymphocyte subsets is very complicated and thus poorly studied. The same is true for the cross-talk between NK cells and ILC3. Although there are some indications that NK cells and ILC3 can indeed interact in homeostatic conditions, as the lack of ILC3-derived lymphotoxin impacted NK cell maturation in the bone marrow [118], there is no evidence reporting that ILC3 could indeed cross-talk with NK cells in the tumor microenvironment. However, since ILC3 has been shown to play an important role in the formation of tumor lymphoid-like stroma and tumor vessels in a murine model of melanoma [119,120], ILC3 could potentially indirectly impact NK cells antitumoral functions by acting on the non-hematopoietic compartment. In contrast, the cross-talk between NK cells and ILC2 has been more conclusively demonstrated in pulmonary metastases. Schuijs et al. showed that IL-33-activated ILC2 suppressed both IFNγ production and cytotoxicity of pulmonary NK cells, leading to inefficient tumor clearing and increased metastatic burden (Figure 2F). The ILC2-dependent suppression of NK cells was indirect and mediated by IL-5-induced lung eosinophilia that affected NK cell fitness [121]. Interestingly, the dampening of NK cell functions mediated by ILC2-induced eosinophilia was not due to transcriptional regulation of NK cell effector molecules but rather to the restraining of NK cell glucose metabolism. Indeed, IL-33 promoted the acidification of the tumor microenvironment, leading to the reduction of glucose. Importantly, the targeting of the ILC2/eosinophils axis by anti-IL-33 or anti-IL-5 treatments restored the NK cell-mediated control of the metastatic burden. These findings not only demonstrated that other innate lymphoid cell populations could indeed indirectly impact NK cell antitumoral functions by shaping the tumor microenvironment, but it also shed new light on the importance of NK cell metabolism for the efficacy of NK cell-mediated antitumor immunity. Whether this networking between ILC2 and NK cells also plays a role in primary tumors has not yet been addressed.

## 5. Conclusions

Although NK cells are very efficient in recognizing and eliminating circulating tumor cells, the capability of NK cells to fight against primary tumors is strongly reduced. NK cells have difficulties in infiltrating and persisting in the tumor microenvironment, and the few NK cells able to reach the tumors are then suppressed by tumor cells. This argues for a reduced contribution of NK cells in the resistance to primary tumors compared to metastasis. However, recent works demonstrated that it is indeed possible to unleash NK cell antitumoral functions and to arm NK cells to fight against primary tumors. The development of therapeutic tools aimed to improve NK cell recognition of tumor cells or to neutralize NK cell immune checkpoints opened a new perspective for enabling NK cell-dependent antitumor immunity in primary tumors and therefore enhanced the relevance of NK cells in cancer.

Tissues determinants orchestrate the activity of NK cells in tissue, and it became clear that in pathological states, the microenvironmental cues shape the immune responses [84,122,123]. The identification of genetic programs and gene signature associated with innate lymphocytes in cancer just started, but it is already evident that this kind of analysis will extend our understanding of antitumor immunity. The comparison of tumor-infiltrating NK cell signature in mice and humans will also reduce the gap between preclinical and clinical studies, favoring the identification of conserved NK cell molecules we can use as new targets for improving the ability of NK cells to control tumor progression.

Immune circuits are crucial to empower NK cell antitumoral functions. We already learned from metastatic tumors that innate immune cells could activate NK cells’ antitumoral functions, and this cross-talk can be boosted by external agents. Extending our knowledge on the “NK cell-centric immune networking” in the primary tumor will allow us to find more accurate ways to shape the tumor microenvironment and to ensure the long-lasting effect of immunotherapies.
